# The effectiveness of a nation-wide implemented fall prevention intervention in the Netherlands in reducing falls and fall-related injuries among community-dwelling older adults with an increased risk of falls: a randomized controlled trial

**DOI:** 10.1186/s12877-025-06967-6

**Published:** 2026-01-24

**Authors:** Maaike van Gameren, Paul B. Voorn, Daniël Bossen, Sanne W.T. Frazer, Judith E. Bosmans, Bart Visser, Mirjam Pijnappels

**Affiliations:** 1 Department of Human Movement Sciences, Faculty of Behavioural and Movement Sciences, Amsterdam Movement Sciences Research Institute, Vrije Universiteit Amsterdam, Van der Boechorststraat 9, Amsterdam, 1081 BT the Netherlands; 2 Faculty of Health, Sport and Physical Activity, Centre of Expertise Urban Vitality, Amsterdam University of Applied Sciences, Tafelbergweg 51, Amsterdam, 1105 BD the Netherlands; 3 Consumer Safety Institute (VeiligheidNL), Overschiestraat 65, Amsterdam, 1062 XD the Netherlands; 4 Department of Health Sciences, Faculty of Science, Amsterdam Public Health Research Institute, Vrije Universiteit Amsterdam, De Boelelaan 1085, Amsterdam, 1081 HV the Netherlands

**Keywords:** Accidental falls, Ageing, Intervention studies, Implementation, Physical therapy

## Abstract

**Background:**

Fall prevention programs have been proven effective in reducing falls and fall-related injuries in specific target groups and settings. However, implementing these programs on a larger scale often requires adjustments for feasibility. This study assessed the effectiveness of the nationally implemented In Balance fall prevention intervention compared to usual care in community-dwelling older adults.

**Methods:**

In this single-blinded randomized controlled trial, 264 non- and pre-frail adults of 65 years or older with an increased fall risk were recruited from eleven centers. The intervention group followed an adapted nation-wide 14-week group In Balance program, including educational sessions and Tai Chi-based balance and strength exercises, delivered by trained therapists. The control group received general physical activity recommendations. Primary outcomes were the number of falls and fall-related injuries over 12 months, recorded via fall diaries and follow-up calls. Secondary outcomes included balance, mobility, and general health. Data were analysed using generalized linear- and mixed-effects models, with multiple imputation for missing data. To obtain a difference in the number of falls per year between the intervention and control groups of 50%, 106 participants per group were required, increased to 264 to account for dropout.

**Results:**

The mean age was 75.2 (SD 5.6) years in the intervention and 75.7 (SD 5.1) years in the control group (*p* > 0.05). The mean number of falls per person over 12 months was not statistically different between the intervention and control group (1.67 (SE 0.24) and 1.98 (0.37), respectively; incidence rate ratio 0.85 (95% CI 0.51–1.43)), nor the mean number of fall-related injuries (0.70 (SE 0.11) and 0.97 (0.18), respectively; incidence rate ratio 0.73 (95% CI 0.44–1.19)). Secondary outcomes also showed no significant differences between groups, frailty status and over time. Attendance averaged 15.5 of 24 sessions.

**Conclusions:**

The adapted In Balance program did not significantly reduce falls, injuries, or improve secondary outcomes compared to usual care. The implemented In Balance program appears to be less effective than a priori assumed, possibly due to limited adherence in practice or insufficient frequency and duration of the program.

**Trial registration:**

Research with human participants: NL9248 (registered February 13 2021, URL: https://www.onderzoekmetmensen.nl/nl/trial/26195).

**Supplementary Information:**

The online version contains supplementary material available at 10.1186/s12877-025-06967-6.

## Background

About 30% of the adults of 65 years or older experience at least one fall per year [1]. Of them, 20% causes potentially life-threatening injuries [2] and about 50% suffers from fear of falling [3]. Specifically in the Netherlands, 33% of the adults of 65 years or older fall at least once per year, of which 10% require treatment at an emergency department. The direct medical costs after a fall were estimated at 1,4 billion euros in 2023 [4]. The consequences of falls are associated with decreased quality of life and high healthcare and societal costs [5]. The number of falls, fall-related injuries and associated healthcare costs are expected to rise further due to the ageing population [4]. The alarming repercussions of falls underscore the essential need for preventing falls and fall-related injuries [6, 7].

Fall prevention interventions have been proven to reduce the number of falls and injuries in older adults [8]. However, the practical implementation of these interventions often encounters barriers that may negatively affect effectiveness [9]. As a result, programs are adjusted when implemented in practice, for example by adapting the frequency and duration of sessions, a more diverse target population, and inconsistent aftercare, which have been associated with reduced effectiveness [10–12]. Furthermore, challenges such as lack of resources, insufficient training for therapists, and resistance from participants can hinder the successful implementation of these interventions [10, 12, 13]. These practical barriers may impact the effectiveness of fall prevention interventions, highlighting the importance of re-evaluating the effectiveness of implemented fall prevention interventions.

An example of such an implemented fall prevention intervention in the Netherlands is the In Balance intervention, which is based on the core elements of fall prevention, including balance and strength training, education on fall risk, home environment modifications, and promoting continuity of exercise [14–16]. In 2006, Faber and colleagues showed that the 20-week precursor to the current In Balance intervention, conducted in a residential care setting, led to a 61% reduction in fall risk among pre-frail older adults in a residential care setting [17]. However, since its initial evaluation, several modifications have been made to the program including a shift in target population from frail to primarily community-dwelling non- and pre-frail older adults, as well as a reduction in program duration from twenty to fourteen weeks. The In Balance program is one of the three fall prevention exercise interventions implemented within the fall prevention chain approach of the Dutch healthcare system [18].

While prior research has demonstrated the effectiveness of the original program, it remains unclear whether these adaptations have influenced its impact in real-world settings. A re-evaluation of the effectiveness of the In Balance program can provide insights into whether and how to further implement and utilise such a fall prevention program as effectively as possible. Therefore, this study aimed to assess the effectiveness of the modified In Balance fall prevention intervention compared to written general physical activity recommendations on the number of falls and fall-related injuries among community-dwelling adults of 65 years or older with an increased fall risk.

## Methods

### Study design and setting

This study was a single-blinded, multicentre randomized controlled trial conducted at various locations across the Netherlands. Eleven centres participated in this study. The In Balance intervention provided by physiotherapists and exercise therapists was assumed similar across sites, since all therapists followed the same education to become an In Balance therapist. The study protocol of this randomized controlled trial has been extensively described elsewhere [19]. Also the accompanying cost-effectiveness analysis and process evaluation are reported elsewhere [20, 21]. The report of the trial follows the recommendations of the CONSORT 2010 Statement [22].

### Participants

The target population consisted of community-dwelling adults aged 65 years or older with an increased fall risk, defined by having experienced at least two falls in the past twelve months and/or having difficulties with moving, walking, or balance [23]. All participants were required to independently execute activities of daily living and walk 100 m. Participants were classified as non-frail or pre-frail based on the phenotype concept by Fried and colleagues [24]. Frail participants were excluded from participation in this study, since the study of Faber and colleagues showed that this group had an increased fall risk after following the intervention [17]. The target group of the In Balance intervention in the Netherlands shifted towards the non- and pre-frail population, but it is unknown for which of these two frailty statuses the intervention is most suitable [17]. The frailty phenotyping includes measures of weight loss, weak grip strength, exhaustion, slow gait speed and low physical activity, see the protocol paper for an elaboration how frailty status was determined [19, 24]. Participants were considered as non-frail if they met none of the criteria, and classified as pre-frail when meeting 1 or 2 of the criteria. If participants met 3 or more of the criteria, they were classified as frail and excluded from participation in this study [24]. Potential participants were also excluded if they participated in a fall prevention intervention in the past 6 months, if they were unable to read and understand Dutch, if they had a severe cognitive impairment defined as a score of 18 or lower on the Mini-Mental State Examination [25], or if they had any self-reported uncontrolled comorbid conditions or contraindications for executing the physical exercises that are part of the In Balance intervention (e.g., cardiovascular, neurological and orthopaedic problems).

The study was conducted according to the guidelines of the Declaration of Helsinki, and approved by the Medical Ethical Committee Brabant (project number P2055) on 10 February 2021. All participants signed informed consent and were aware that participation is voluntary.

### Recruitment, randomization, stratification, blinding and treatment allocation

Participants were recruited through flyers, advertisements in local newspapers, and the network of In Balance therapists. Randomization was conducted in a 1:1 ratio between the intervention and control groups, with stratification based on frailty status. The allocation sequence was generated online via Sealed Envelope [26]. To ensure randomization, sealed envelopes were prepared in blocks of 10, with five envelopes per group. One of the researchers assigned participants to a group by opening a sealed envelope. All investigators and assessors involved in the study remained blinded to group assignment. However, due to the nature of the study, blinding of participants and therapists was not possible.

### Intervention and control groups

The In Balance intervention is a fourteen-week group intervention for older adults at risk of falls and was provided by registered and certified physical therapists and exercise therapists [27]. The intervention consists of three phases. The first phase (week 1) comprises one information meeting. In the second phase (week 2–4), there are three weekly educational meetings. The third phase (week 5–14) consists of a physical exercise programme with two one-hour training sessions per week. Exercises integrate principles of Tai Chi, focusing on balance and strength. For a more elaborate description of the In Balance intervention, see Additional file 1 and the study protocol of this study [19].

The control group received usual care, supplemented with a flyer containing general physical activity recommendations based on the World Health Organization’s guidelines [28]. This flyer provided guidance on physical activity, strength, and balance for older adults, but did not constitute structured exercise and was intended as a minimal, passive resource in addition to routine care.

### Data collection and outcome measures

#### Primary outcome measures

The primary outcome measures were the number of falls and fall-related injuries over 12 months. A fall was defined as coming unintentionally to the ground or a lower level [29]. These were assessed with fall diaries, including questions on the causes, circumstances and consequences of falls and monthly telephone calls for the follow-up on the fall diaries [30, 31].

#### Secondary outcome measures

Secondary outcome measures were assessed at three time points; at entry of the study (baseline, M0), after 4 months to determine the short-term effects (M4) and after 12 months to determine the long-term effects (M12).

Demographic characteristics were assessed at M0. General health status was self-reported using the Physical Functioning domain and Emotional Wellbeing domain of the 36-Item Short Form Health Survey (SF-36) at M0, M4, and M12 [32] which was administered online or on paper based on the preferences of the participant. Both domains are on a scale of 0 to 100, where 0 represents the least and 100 the most favourable outcome [33].

At each time point, we invited participants for physical tests and measurements to a location in their neighbourhood. The Four Stage Balance Test was used to assess static balance through four progressively challenging standing positions—feet together, semi-tandem, tandem, and single-leg stance—with the score ranging from 0 (lowest) to 40 (highest), based on the ability to hold each position for 10 s [34]. The Timed Up and Go (TUG) test was used to measure functional mobility by timing how long it takes a person to stand up from a chair, walk three meters, turn around, walk back, and sit down, with lower times indicating better performance [35].

The tests have a moderate (FSBT) to excellent (TUG and SF-36) reliability [36–38].

### Other measures

Adherence to the In Balance intervention was monitored by keeping attendance lists by the In Balance therapists. Physical activity was assessed with an inertial sensor (DynaPort MoveMonitor Plus, McRoberts BV, The Netherlands) at M0, M4, and M12 [39, 40]. The sensor was worn on the lower back for seven consecutive days, preferably day and night, except during water activities, and was returned afterwards by mail. The mean number of hours being physically active per day was determined.

### Statistical analysis

Data was analysed using RStudio (Version 2023.06.0). All analyses were performed according to the intention-to-treat principle. Moreover, a per-protocol analysis was done comparing individuals of the In Balance group who attended at least 75% of all In Balance sessions with those of the control group who did not participate in any fall prevention intervention during the follow-up [41].

### Sample size

In the general population of older adults, about one in three persons falls at least once per year [42]. To obtain a difference in the number of falls per year of 50% less falls in the intervention than in the control group, a minimum of 106 persons were required per group at a power of 0.80, beta of 0.20 and alpha of 0.05 (two-sided). Taking into account a dropout rate of 20%, the total required sample size was a minimum of 256 participants. Because the optimal number of participants in an In Balance training group is 12, we included a total of 264 participants. The sample size calculation was performed using G*Power, based on an expected between-group difference of 50% in the number of falls, to detect a meaningful reduction in fall incidence [43].

### Demographic characteristics

Normality of the data was assessed by visual inspection of histograms and Q–Q plots, and confirmed using the Shapiro–Wilk test. Data were described using means and standard deviations for normally distributed continuous variables, medians and interquartile ranges (IQR) for non-normally distributed continuous variables, and numbers and percentages for non-continuous variables. Differences between the intervention and control group were tested by a two-sided t-test (alpha = 0.05) for normally distributed data and Mann-Whitney U test for non-normally distributed data. To check for potential selection bias or systematic drop-out, characteristics of participants with complete data were compared to those with missing data.

### Missing data imputation

To prevent bias due to selective missingness, we imputed both the primary and secondary outcomes using Multiple Imputation by Chained Equations (MICE) with Predictive Mean Matching using the R ‘MICE’ package [44]. The number of falls and injuries were imputed per four months. The secondary outcomes were imputed at M0, M4, and M12. The number of datasets was increased until the fraction of missing information was less than 5%, resulting in 20 imputed datasets. Analyses were done on the imputed datasets and results were pooled thereafter using Rubin’s rules [45].

### Primary and secondary outcomes

The effectiveness of the In Balance intervention in comparison with the control group was analysed using generalized linear models for the primary outcomes, and linear mixed-effects models for the secondary outcomes. We included age and sex as confounders in all analyses [46] and physical activity was included as confounder in the analyses of the primary outcomes [16]. Other variables were considered confounders if the estimate for the randomization group changed by at least 10% after adding that variable to the model.

The number of falls and injuries were analysed using generalized linear models with a negative binomial regression to account for their count distribution. In case the Intraclass Correlation Coefficient (ICC) was 0.2 or higher derived from a linear mixed-effects model, therapist was included as random effect in the models [47]. Despite the standardized training of therapists, the context of different sites of a widely implemented intervention may affect outcomes. To check for this, we calculated the Intraclass Correlation Coefficient (ICC) between therapists. In case the ICC was 0.2 or higher, therapist was included as random effect in the models. However, we found a low ICC (about 0.01), indicating that the data is largely independent of the therapist, and therefore correction for therapist was not needed [29]. The mean number of falls and fall-related injuries, incidence rate ratios (IRR) and 95% confidence intervals (CI) were reported. Faller status (no falls or injuries vs. at least one fall or injury) and number needed to treat to prevent a fall or fall-related injury were also described.

The Timed Up and Go test, Four Stage Balance test, and the Physical Functioning domain and Emotional Wellbeing domain of the SF-36 were analysed with linear mixed-effect models [32, 34, 35]. We included participant as a hierarchical level in the models to account for the longitudinal nature of the data. The mean scores of the secondary outcome measures, Relative Effects, and 95% confidence intervals were reported.

To identify possible differences in intervention effects between non-frail and pre-frail phenotypes, we performed an a priori defined analysis, stratified for frailty status (non- and pre-frail). These analyses were conducted in the same way as described above for the main analysis, but separately for the non-frail and pre-frail groups [41].

## Results

**Fig. 1:**
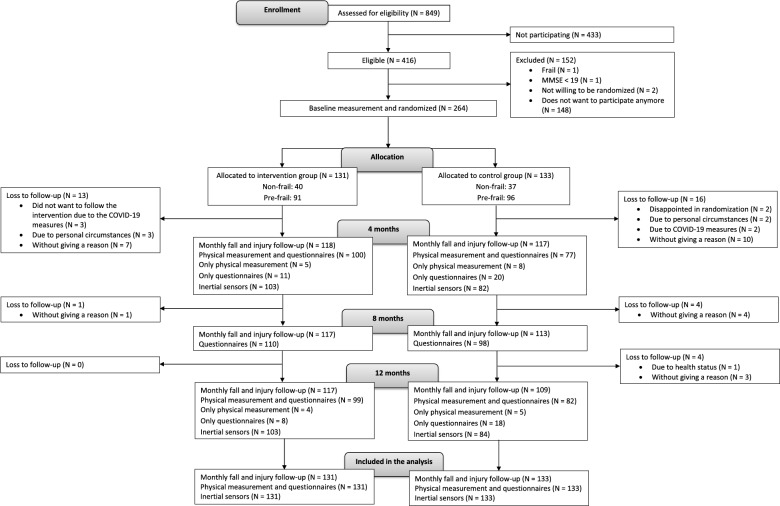
Flowchart of the study recruitment and follow-up. Numbers decrease due to drop-outs, but after imputation, the original numbers are restored as all missing data were imputed

Between June 2021 and January 2023, we screened 849 potential participants (Fig. 1). Of these, 264 people were included in the study between August 2021 and January 2023 and randomly assigned to the intervention group (*n* = 131) or the control group (*n* = 133). Follow-up finished in January 2024. We obtained complete falls and injuries data of 117 persons in the intervention group, and 106 persons in the control group. The In Balance program was provided by 15 therapists in 9 municipalities.

Table 1 presents the baseline characteristics of the participants. Education was the only baseline characteristic that differed significantly between the intervention and control group (*p* = 0.02). Drop-out comparison analyses showed that participants with complete follow-up had significantly higher baseline MMSE scores and reported fewer prior falls compared to those with incomplete follow-up.

**Table 1:** Baseline characteristics of the participants. Characteristics are presented as n (%) unless specified otherwise

Variable	In Balance group (*N* = 131)	Control group (*N* = 133)
Age (years) (median (IQR))	74.9 (70.7–79.5)	75.3 (71.2–79.8)
Sex (female)	99 (76%)	102 (77%)
Body Mass Index (kg/m^2^) (median (IQR))	25.6 (23.7–29.6)	26.4 (23.3–28.7)
Mini-Mental State Examination (score) (median (IQR))	28 (27–29)	28 (27–29)
Marital status		
Lawfully married/living together Unmarried/divorced/widowed	58 (45%) 70 (55%)	61 (50%) 61 (50%)
Having children (yes, n (%))	96 (75%)	92 (74%)
Education^1^		
Low Moderate High	0 (0%) 38 (30%) 90 (70%)	7 (6%) 31 (25%) 86 (69%)
Smoking (yes, n (%))	6 (5%)	7 (6%)
Use of alcohol (yes, n (%))	91 (71%)	92 (74%)
Use of different medications per week (median (IQR))	2 (1–4)	3 (0–4)
Dizziness (yes, n (%))	36 (28%)	27 (23%)
Incontinence (yes, n (%))	58 (45%)	65 (52%)
Number of falls in previous year		
None Once Twice or more	35 (28%) 35 (28%) 56 (44%)	35 (28%) 37 (30%) 52 (42%)
Use of aids (yes, n (%))		
Walking Vision^2^ Hearing	23 (18%) 126 (98%) 27 (21%)	19 (15%) 118 (95%) 30 (24%)
Physical activity per day (median (IQR))		
Number of hours being physically active Number of steps	1.4 (0.9–1.7) 6312 (4128–8267)	1.4 (1.0-1.7) 6229 (4402–8303)

^1^Low = primary school, moderate = Lower vocational education/Lower general secondary education/Trade school/Domestic science school/Extended primary education, high = Higher general secondary education/Pre-university education/Secondary school for girls/Higher civic school/Secondary vocational education/Higher professional education/University

^2^ Visual aids include reading glasses, glasses to see far, multifocal glasses, magnifying glass, or other

### Effects on primary outcome *–* Falls and fall-related injuries

In the intervention group, 81 (61.8%) participants experienced at least one fall over 12 months follow-up, while in the control group this were 88 (66.2%) participants. The incidence rate ratio (IRR) was 0.85 (95% CI: 0.51–1.43) indicating that the number of falls in the intervention group was 15% less than in the control group (Table 2). The number needed to treat to prevent one fall was 3.32. In the intervention group, 56 (42.7%) participants experienced at least one fall-related injury. The IRR was 0.73 (95% CI: 0.44–1.19) (Table 2). The number needed to treat to prevent one injury was 3.69. Most results did not reach statistical significance; we only found a significant effect of the intervention in the IRR for fall-related injuries in the non-frail group (*p* = 0.02).

**Table 2:** The mean number and incidence rate ratio of falls and fall-related injuries

	Intervention group (mean (SE))	Control group (mean (SE))	IRR crude analysis (95% CI)	IRR adjusted for confounders^1^ (95% CI)	IRR adjusted for confounders (95% CI)
**Main analysis**	*n* = 131	*n* = 133			
Mean number of falls	1.67 (0.24)	1.98 (0.37)	0.85 (0.52;1.42)	0.85 (0.52; 1.42)	0.85 (0.51; 1.43)^2^
Mean number of injuries	0.70 (0.11)	0.97 (0.18)	0.73 (0.44; 1.20)	0.73 (0.44; 1.19)	0.73 (0.44; 1.19)^1^
**Stratified for frailty status**					
*Non-frail*	*n* = 40	*n* = 37			
Mean number of falls	1.40 (0.24)	1.76 (0.37)	0.80 (0.46; 1.40)	0.79 (0.45; 1.37)	0.88 (0.51; 1.53)^3^
Mean number of injuries	0.38 (0.11)	0.94 (0.23)	0.40 **(0.18; 0.89)**	0.39 **(0.18; 0.87)**	0.39 **(0.18; 0.87)**^**1**^
*Pre-frail*	*n* = 91	*n* = 96			
Mean number of falls	1.79 (0.32)	2.06 (0.45)	0.88 (0.48; 1.61)	0.87 (0.48; 1.60)	0.82 (0.46; 1.49)^4^
Mean number of injuries	0.84 (0.14)	0.98 (0.21)	0.86 (0.49; 1.52)	0.86 (0.49; 1.51)	0.82 (0.46; 1.46)^5^
**Per-protocol**	*n* = 54	*n* = 130			
Mean number of falls	1.84 (0.37)	1.96 (0.38)	0.95 (0.57; 1.60)	0.98 (0.57; 1.68)	0.88 (0.52; 1.47)^6^
Mean number of injuries	0.72 (0.17)	0.96 (0.18)	0.76 (0.43; 1.35)	0.76 (0.42; 1.35)	0.62 (0.34; 1.12)^7^

The mean number of falls and injuries are presented per person per year

IRR = Incidence Rate Ratio: intervention group regarding control group. The incidence rate ratio (IRR) was calculated by exponentiating the estimated coefficients from the negative binomial regression model

Table showing the mean number of falls and injuries, which equals the fall and injury rate, as all missing data has been imputed

^1^ Analysis adjusted for age, sex and physical activity, ^2^ Analysis adjusted for age, sex, physical activity, smoking, incontinence, number of falls in year before participation in study, walking aid and physiotherapy, ^3^ Analysis adjusted for age, sex, physical activity, Mini-Mental State Examination, marital status, number of falls in year before participation in study, use of vision aids and use of walking aid, ^4^ Analysis adjusted for age, sex, physical activity, Mini-Mental State Examination, education, smoking, incontinence, number of falls in year before participation in study, use of walking aid and physiotherapy, ^5^ Analysis adjusted for age, sex, physical activity, Mini-Mental State Examination, having children, incontinence, number of falls in year before participation in study, use of vision aid, use of walking aid and physiotherapy ^6^ Analysis adjusted for age, sex, physical activity, Body Mass Index, Mini-Mental State Examination, living alone, education, smoking, use of alcohol, dizziness, incontinence, number of falls in year before participation in study, use of vision aid, use of hearing aid, use of walking aid and physiotherapy, ^7^ Analysis adjusted for age, sex, physical activity, dizziness, number of falls in year before participation in study, use of walking aid and physiotherapy

CI in bold means *p* < 0.05

### Effects on secondary outcomes of the four stage balance test, timed up and go test and 36-Item short form health survey

**Fig. 2:**
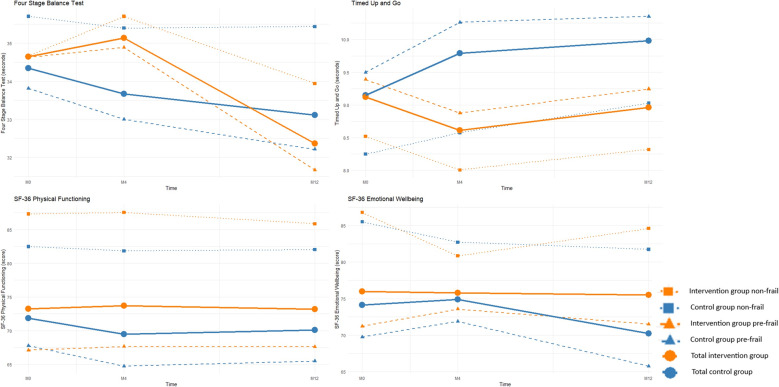
Trends of the secondary outcomes

Figure 2 shows the course of the Four Stage Balance Test, Timed Up and Go Test and SF-36 outcomes over the follow-up period. We found no significant differences in any of these outcomes at M4 and M12 compared to M0 between the intervention and control groups (Table 3).

**Table 3:** The mean scores over time and relative effects of the secondary outcomes

	Intervention group (mean (SE)) (*n* = 131)			Control group (mean (SE)) (*n* = 133)			RE crude analysis (95% CI)	RE crude analysis (95% CI)	RE crude analysis (95% CI)	RE adjusted for confounders^1^ (95% CI)	RE adjusted for confounders^1^ (95% CI)	RE adjusted for confounders^1^ (95% CI)
	M0	M4	M12	M0	M4	M12	M0 – M4	M0 – M12	Overall effect	M0 – M4	M0 – M12	Overall effect
Four Stage Balance Test (s)	34.6 (0.58)	35.1 (0.67)	32.4 (0.83)	34.3 (0.58)	33.7 (1.22)	33.1 (1.15)	1.17 (-1.69; 4.02)	-1.04 (-3.97; 1.88)	-0.31 (-2.44; 1.82)	1.17 (-1.69; 4.03)	-1.05 (-3.97; 1.88)	-0.31 (-2.44; 1.82)
Timed Up and Go Test (s)	9.1 (0.21)	8.6 (0.29)	9.0 (0.27)	9.2 (0.22)	9.8 (0.78)	10.0 (0.55)	-1.16 (-2.85; 0.54)	-1.00 (-2.32; 0.33)	-1.05 (-2.07; -0.03	-1.16 (-2.85; 0.54)	-1.00 (-2.32; 0.33)	-1.05 (-2.07; -0.03)
36-Item Short Form Health Survey Physical functioning Emotional wellbeing	73.2 (1.78) 76.0 (1.32)	73.7 (3.21) 75.8 (1.94)	73.2 (2.27) 75.5 (1.92)	71.8 (1.69) 74.1 (1.30)	69.5 (3.94) 74.9 (2.65)	70.1 (3.97) 70.2 (3.53)	2.82 (-8.12; 13.76) -0.93 (-6.84; 4.98)	1.70 (-7.24; 10.64) 3.47 (-4.90; 11.84)	2.07 (-4.66; 8.81) 2.00 (-3.68; 7.68)	2.82 (-8.12; 13.76) -0.93 (-6.84; 4.98)	1.70 (-7.24; 10.64) 3.47 (-4.90; 11.84)	2.07 (-4.66; 8.81) 2.00 (-3.68; 7.68)

RE = Relative Effect: intervention group regarding control group. The RE is calculated as the ratio of the change from baseline to the respective time point (M4 or M12) in the intervention group compared to the control group. The overall RE is computed as a weighted average of the REs at M4 and M12, proportional to the duration of each period

^1^ Analysis adjusted for age and sex

### Effects on analyses stratified for frailty status and per-protocol

**Fig. 3:**
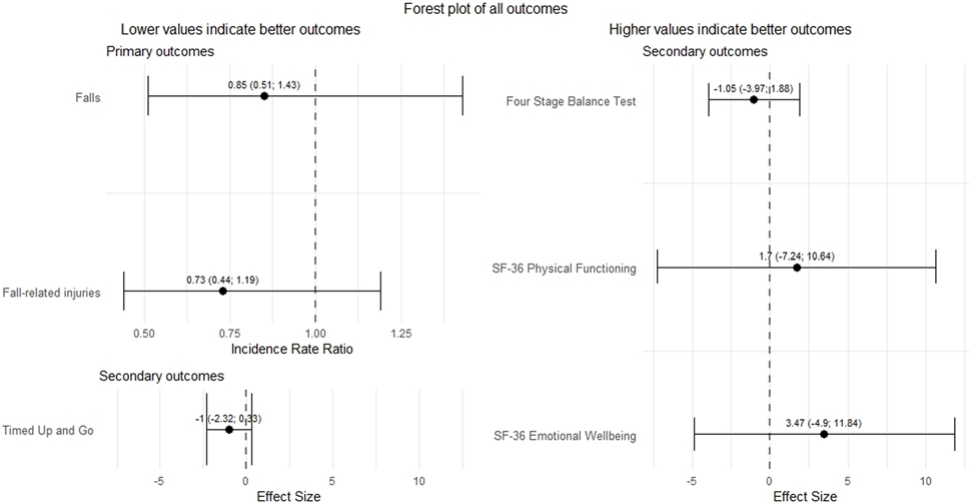
Forest plot showing an overview of all outcome measures. RE = Relative Effect: intervention group regarding control group. The RE is calculated as the ratio of the change from baseline to the respective time point (M4 or M12) in the intervention group compared to the control group. The overall RE is computed as a weighted average of the REs at M4 and M12, proportional to the duration of each period

Additional file 2 shows baseline characteristics stratified for intervention group and frailty status, and Additional file 3 shows baseline characteristics stratified for adherence to the protocol. Table 2 shows the mean number and incidence rate ratio of falls and fall-related injuries of the intervention group and control group stratified for frailty status and according to a per-protocol analysis. No significant differences were found between the intervention and control group, except for the mean number of injuries in the non-frail group (*p* = 0.02). Additional file 4 shows the results of the analyses of the secondary outcomes stratified for frailty status, and Additional file 5 of the per-protocol analyses. Again no significant differences were found for these secondary outcomes, except for the RE for the overall effect of the TUG for the per-protocol analysis (ES: -1.37, 95% CI: -2.60 to -0.14, p = 0.03). For an overview of all outcome measures, see Fig. 3.

### Process outcomes

#### Adverse events

No severe adverse events were reported that were related to the intervention or control group or the measurements during this study.

#### Intervention adherence

Participants attended a mean of 15.5 out of the 24 In Balance sessions (64.6%), including study drop-outs. Of the 131 participants randomized in the intervention group, 15 participants (11.5%) dropped out of the In Balance intervention. Excluding the intervention drop-outs, the mean number of attended sessions was 17.9 (74.6%).

## Discussion

In this effectiveness analysis of the In Balance intervention program, as implemented routinely in non- and pre-frail community-dwelling older adults, we observed statistically non-significant differences in the number of falls, fall related injuries, balance, mobility, and quality of life, except for the mean number of fall injuries in the non-frail group and the TUG in the per-protocol analysis. However, given the large number of non-significant findings, these results should be interpreted with caution. Nonetheless, all differences were in favor of the intervention group. Analyses stratified for frailty and per-protocol analyses showed similar results.

There are various possible explanations for these findings. First, although the core elements of effective fall prevention programs were maintained in the In Balance intervention, the shortened duration (14 weeks) with unaltered frequency (two times per week) of the program might be insufficient to achieve the desired health effects within this time frame [7]. Reviews suggest that fall prevention interventions should comprise at least 26 weeks to obtain significant effects [7]. Extending the intervention is currently not feasible within the Dutch health care system, but there is a pathway for transition into regular follow-up exercise programs [18]. Therefore, the In Balance intervention can serve as a stepping-stone toward long-term effects on physical activity and fall prevention, provided that exercises are continued. The process evaluation of the In Balance intervention showed that some municipalities offered follow-up exercise programs, but most did not yet [20].

Second, low adherence to the In Balance intervention may have also contributed to the lack of significant results, with an average of 15.5 out of 24 sessions (65%) attended. However, the per-protocol analysis also showed no significant effect of the In Balance intervention. The 65% adherence we found in this study is in line with a meta-analysis that found an average adherence of 66% in fall prevention programs, while 80% adherence is recommended for fall prevention programs including exercise [11, 48]. In the per-protocol analysis of this study, the adherence was 83% of attending all sessions.

Comparing our study with the effectiveness study of the precursor of the program by Faber and colleagues, both showed that the In Balance fall prevention program can affect fall rates and functional performance, yet with differing outcomes [17]. Faber and colleagues observed higher fall risk among frail participants and improvements in pre-frail individuals. Their study was conducted in an institutional setting, and additionally, the target group was later extended to non- and pre-frail older adults. Based on the results of Faber and colleagues, we expected that the non-frail population would benefit even more from the In Balance intervention. In contrast, our study showed no significant reduction in falls or fall-related injuries compared to usual care, nor significant differences in secondary outcomes like balance and mobility and between non- and pre-frail older adults. These discrepancies between the study of Faber and colleagues and our study could be attributed to differences in target group, setting, and program duration. Another explanation for the smaller effects observed in the current study compared to the study of Faber and colleagues may be the lower overall exercise volume of the modified In Balance program. Although the session frequency was maintained, the shorter duration of the program resulted in fewer total training sessions (24 versus 36) [17]. As a higher exercise volume is generally associated with greater improvements in balance and strength, this may have limited the potential effect of the program [49]. It is therefore plausible that maintaining the original total exercise volume could have yielded stronger or even significant results. Therefore, adherence to the intervention is recommended, because attending more sessions is expected to yield greater benefits. Similarly, maintaining regular physical activity after the intervention may help to sustain the improvements that have been achieved [49]. For example, the institutional setting in Faber’s study might have offered a more controlled environment for delivering the program, which could lead to better adherence and more consistent implementation, potentially enhancing the intervention’s effectiveness. In the Netherlands, fall risk is assessed based on several factors, including whether a person has fallen in the past 12 months, concerns about falling, difficulties with movement, walking, or balance, and the 4-meter walk test as an objective measure of fall risk [23, 50, 51]. With this broad approach, In Balance is not only aimed at those with a high risk of falling but also at individuals with an increased risk, for whom preventive measures can help reduce the likelihood of future falls.

This raises the question what the most appropriate target population of the In Balance intervention is. According to recommendations from a review of Sherrington and colleagues, fall prevention interventions should be targeted at older adults in the general community as well as community-dwelling older adults with an increased risk of falls [49]. Our study findings align with these recommendations. Specifically, we observed that the risk of falls was non-significantly lower with approximately 20% among non-frail individuals who participated in the intervention compared to those who did not, in contrast with a 12% lower risk of falls among pre-frail participants. These results highlight the benefits of also targeting non-frail older adults with the In Balance program.

There was a 15% difference in the mean number of falls between the intervention and control groups, whereas earlier studies showed a reduction of 17% to 39% in the number of falls after exercise interventions, depending on the type of exercise (balance, functional, and resistance exercises) and the delivery method (effect is higher if there is more than 3 h of exercise per week for at least 26 weeks) [7]. Approximately 62% of the intervention group and 66% of the control group, consisting of older adults with increased fall risk, experienced at least one fall during the one-year follow-up, which is considerably higher than the 33% reported in the literature for the general population of older adults, and also higher than the 19% found in a study within the Canadian Longitudinal Study on Aging of 2022 [52]. This could possibly be explained because this study monitored falls by asking the question whether a participant fell in the past 12 months instead of prospective monitoring of falls. The intensive follow-up with fall diaries and monthly telephone calls likely resulted in more accurate reporting, which could explain the higher percentages compared to other studies. Moreover, depending on the population, between 20% and 60% of older people suffer from fall-related injuries [53, 54]. In our study we found that 43% of participants in the In Balance group and 50% of those in the control group suffered from a fall-related injury, which is relatively high and in line with previous studies.

The strength of this study is that the In Balance intervention was already implemented in practice, allowing us to evaluate its effectiveness in a real-world setting. This pragmatic design enhances the external validity of our findings, as it reflects the actual conditions under which the intervention is delivered. Additionally, using robust methods to reduce bias, such as blinded outcome assessment and intention-to-treat analysis, strengthens the credibility of our results. Furthermore, in a complete case sensitivity analysis, the results were consistent with those of the main analysis, confirming the robustness of our findings. Last, this study included a wide range of outcome measures, including physical activity measures.

A limitation of this study is that it was powered based on an anticipated 50% less falls in the intervention group compared to the control group, but the observed reduction was only 15%, making the study underpowered for the primary outcomes. Second, the primary outcomes—the number of falls and fall-related injuries—were collected through self-reported data. The combination of monthly phone calls and fall and injury diaries has been shown the most accurate method and helped mitigate recall bias [31]. At the same time, intensive monitoring during the post-intervention follow-up period may have led to a high number of reported falls compared to retrospective questionnaires.

Furthermore, this study used an ambitious estimate of 50% less falls in the intervention group compared to the control group for the sample size calculation, while in general exercise interventions decrease the number of falls with about 23% [8]. This assumption was based on the expectation of a substantial effect to ensure clinical relevance. The intention was to detect a meaningful reduction in fall incidence that would support large-scale implementation of the program. Besides, a limitation of this study is that attendance was not distinguished between participation in the information/education sessions (weeks 1–4) and the exercise sessions (weeks 5–14), because this data was not available in most individuals. Last, a limitation of this study is that, although the In Balance intervention includes education for environmental modifications, we did not monitor this data was not available in most individuals. Last, a limitation of this study is that, although the In Balance intervention includes education for environmental modifications, we did not monitor whether participants implemented these changes in their own homes. As a result, we cannot assess the impact of environmental adjustments on fall prevention outcomes.

There are some practical implications for fall prevention programs like In Balance based on the results of this study. First, it may be important to implement follow-up exercise programs after the initial intervention to achieve the desired health outcomes. A fall prevention program, such as In Balance, aims for long term benefits, e.g. to protect people from falls in the long term. To maintain these long-term benefits, it is essential that knowledge and skills are periodically refreshed, and that the training effects gained during the intervention are sustained. Follow-up activities, particularly those involving exercises targeting strength and balance, can play an important role in this. Moreover, in our study, we obtained the largest effects directly after the intervention compared to the long-term effects. Future studies should consider and analyse maintaining an adequate total exercise volume when implementing shorter intervention durations. As suggested by Sherrington and colleagues, sufficient exercise dose is essential to achieve meaningful effects on fall prevention outcomes [49]. Ensuring that the total volume of exercise is preserved, for instance by increasing session frequency and stimulating home exercises is crucial. For example, enhancing participant engagement and self-management through personalized support and regular check-ins could also improve exercise volume, adherence and effectiveness; and may possibly be boosted by telemedicine and technological solutions. Therefore, Further research on the impact of continuous fall prevention and exercise interventions, rather than a stand-alone program is strongly recommended. Additionally, future research should include a larger sample size, as our power analysis was based on 50% less falls in the intervention group compared to the control group, and appeared to be 15%. Further investigations should include tailoring interventions for both non-frail and pre-frail older adults to maximize benefits across a wider population. Last, although this study was conducted in the Netherlands, its findings are valuable for other countries with similar demographic trends. However, differences in how fall prevention interventions are organized should be considered when applying these results in international contexts.

## Conclusions

The implemented In Balance fall prevention intervention resulted in no statistically significant reduction in falls and fall-related injuries. Nonetheless, all differences were in favor of the intervention group. This may be explained by the possibly insufficient adherence, frequency, volume and duration of the program. To achieve long-term benefits from fall prevention interventions, attention should be given to self-management and follow-up activities that encourage participants to continue exercising after the intervention has ended to achieve the desired health outcomes, particularly those involving exercises targeting strength and balance. Further research should explore the impact of continuous fall prevention and exercise interventions, rather than a stand-alone program.

## Supplementary Information

Additional file 1. Trial treatment manual of the In Balance fall prevention intervention.

Additional file 2. Baseline characteristics of the participants stratified for frailty status.

Additional file 3. Baseline characteristics of the participants per-protocol.

Additional file 4. Secondary results stratified for frailty status.

Additional file 5. Secondary results per-protocol.

## Data Availability

The datasets used and analysed during the current study are anonimized available from the corresponding author on reasonable request.

## Abbreviations

BMI: Body Mass Index
CI: Confidence Interval
SF-36: 36-Item Short Form Health Survey
ICC: Intraclass Correlation Coefficient
IRR: Incidence Rate Ratio
M0: Month 0
M4: Month 4
M12: Month 12
MICE: Multiple Imputation by Chained Equations
SE: Standard Error
TUG: Timed Up and Go
