# Supplementary material for: The effectiveness of a nation-wide implemented fall prevention intervention in the Netherlands in reducing falls and fall-related injuries among community-dwelling older adults with an increased risk of falls: a randomized controlled trial
Source: BMC Geriatr. 2026 Jan 24;26:227. doi: 10.1186/s12877-025-06967-6 (PMC12911379; doi:10.1186/s12877-025-06967-6)
Supplement: Supplementary file 1 — Additional file 1. Trial treatment manual of the In Balance fall prevention intervention. [file 12877_2025_6967_MOESM1_ESM.docx]

**Additional file 3: Baseline characteristics of the participants per-protocol**

**Table 1. Baseline characteristics of the participants per-protocol. Characteristics are presented as n (%) unless specified otherwise.**

| **Variable** | **Intervention group (N = 54)** | **Control group (N = 130)** |
| --- | --- | --- |
| Age (years) (median (IQR)) | 75.3 (70.8 – 81.6) | 75.1 (71.1 – 79.7) |
| Gender (female) | 40 (74.1%) | 100 (76.9%) |
| Body Mass Index (kg/m^2^) (median (IQR)) | 25.1 (23.2 – 27.7) | 26.4 (23.3 – 28.7) |
| Mini-Mental State Examination (score) (median (IQR)) | 28 (27 – 29) | 28 (27 – 29) |
| Frailty status (pre-frail) | 41 (75.9%) | 93 (71.5%) |
| Marital status  Lawfully married/living together  Unmarried/divorced/widowed | 26 (48.1%)  28 (51.9%) | 58 (48.7%)  61 (51.3%) |
| Having children | 37 (68.5%) | 90 (74.4%) |
| Living alone | 28 (51.9%) | 66 (54.5%) |
| Education  Low  Moderate  High | 0 (0.0%) 17 (31.5%) 37 (68.5%) | 7 (5.8%) 31 (25.6%) 83 (68.6%) |
| Smoking | 1 (1.9%) | 7 (5.8%) |
| Use of alcohol | 40 (74.1%) | 91 (75.2%) |
| Use of different medications per week (median (IQR)) | 3 (1 – 5) | 3 (0 – 4) |
| Dizziness | 16 (29.6%) | 27 (23.9%) |
| Incontinence | 29 (53.7%) | 62 (51.2%) |
| How often fallen in previous year before start study  None/once  Twice or more | 24 (45.3%) 29 (54.7%) | 70 (57.9%) 51 (42.1%) |
| Use of aids   Walking  Vision  Hearing | 12 (22.2%) 53 (98.1%) 14 (25.9%) | 17 (14.0%) 116 (95.9%) 29 (24.0%) |
| Having physiotherapy | 20 (37.0%) | 38 (31.4%) |
| Physical activity (median (IQR))  Number of hours being physically active  Number of steps | 1.3 (0.9 – 1.6) 5839.6 (4248.2 – 7553.1) | 1.4 (1.0 – 1.7) 6229.2 (4401.9 – 8355.1) |
