# Supplementary material for: The effectiveness of a nation-wide implemented fall prevention intervention in the Netherlands in reducing falls and fall-related injuries among community-dwelling older adults with an increased risk of falls: a randomized controlled trial
Source: BMC Geriatr. 2026 Jan 24;26:227. doi: 10.1186/s12877-025-06967-6 (PMC12911379; doi:10.1186/s12877-025-06967-6)
Supplement: Supplementary file 2 — Additional file 2. Baseline characteristics of the participants stratified for frailty status. [file 12877_2025_6967_MOESM2_ESM.docx]

**Additional file 2: Baseline characteristics of the participants stratified for frailty status**

**Table 1. Baseline characteristics of the participants stratified for frailty status. Characteristics are presented as n (%) unless specified otherwise.**

| **Variable** | **Intervention group Non-frail (N = 40)** | **Intervention group Pre-frail (N = 91)** | **Control group Non-frail (N = 37)** | **Control group pre-frail (N = 96)** |
| --- | --- | --- | --- | --- |
| Age (years) (median (IQR)) | 74.0 (71.5 - 78.3) | 75.3 (70.6 – 80.3) | 75.1 (72.1 – 77.3) | 75.4 (71.2 – 80.5) |
| Gender (female) | 27 (67.5%) | 72 (79.1%) | 26 (70.3%) | 76 (79.2%) |
| Body Mass Index (kg/m^2^) (median (IQR)) | 24.5 (23.1 – 26.5) | 26.3 (23.8 – 30.1) | 25.7 (23.0 – 27.9) | 27.0 (23.6 – 29.1) |
| Mini-Mental State Examination (score) (median (IQR)) | 28 (27 – 30) | 28 (26 – 29) | 28 (26 – 29) | 28 (27 – 29) |
| Marital status  Lawfully married/living together  Unmarried/divorced/widowed | 21 (53.8%)  18 (46.2%) | 37 (41.6%)  52 (58.4%) | 15 (41.7%)  21 (58.3%) | 46 (53.5%)  40 (46.5%) |
| Having children | 29 (74.4%) | 67 (75.3%) | 27 (75.0%) | 65 (73.9%) |
| Living alone | 20 (51.3%) | 52 (58.4%) | 21 (58.3%) | 45 (51.1%) |
| Education   Low  Moderate  High | 0 (0.0%) 9 (23.1%) 30 (76.9%) | 0 (0.0%) 29 (32.6%) 60 (67.4%) | 2 (5.6%) 6 (16.7%) 28 (77.8%) | 5 (5.7%) 25 (28.4%) 58 (65.9%) |
| Smoking | 3 (7.7%) | 3 (3.4%) | 2 (5.6%) | 5 (5.7%) |
| Use of alcohol | 28 (71.8%) | 63 (70.8%) | 28 (77.8%) | 64 (72.7%) |
| Use of different medications per week (median (IQR)) | 1 (0.0 – 3.0) | 3 (1.0 – 4.0) | 2 (0.0 – 3.3) | 3 (1.0 – 4.0) |
| Dizziness | 7 (17.9%) | 29 (32.6%) | 5 (15.2%) | 22 (26.5%) |
| Incontinence | 15 (38.5%) | 43 (48.3%) | 15 (41.7%) | 50 (56.8%) |
| How often fallen in previous year before start study  None/once  Twice or more | 26 (66.7%) 13 (33.3%) | 44 (50.6%) 43 (49.4%) | 23 (63.9%) 13 (36.1%) | 49 (55.7%) 39 (44.3%) |
| Use of aids   Walking  Vision  Hearing | 3 (7.7%) 39 (100%) 7 (17.9%) | 20 (22.5%) 87 (97.8%) 20 (22.5%) | 1 (2.8%) 34 (94.4%) 9 (25%) | 18 (20.5%) 84 (95.5%) 21 (23.9%) |
| Having physiotherapy | 8 (20.5%) | 36 (41.4%) | 10 (27.8%) | 30 (34.1%) |
| Physical activity (median (IQR))  Number of hours being physically active  Number of steps | 1.5 (1.0 – 1.8)  6792.8 (4660.6 – 8434.3) | 1.2 (0.8 – 1.6)  5282.9 (3987.2 – 7822.2) | 1.5 (1.2 – 1.7)  6944.6 (5440.2 – 8866.4) | 1.3 (0.9 – 1.7)  5748.4 (4105.6 – 7945.6) |
