# Supplementary material for: The effectiveness of a nation-wide implemented fall prevention intervention in the Netherlands in reducing falls and fall-related injuries among community-dwelling older adults with an increased risk of falls: a randomized controlled trial
Source: BMC Geriatr. 2026 Jan 24;26:227. doi: 10.1186/s12877-025-06967-6 (PMC12911379; doi:10.1186/s12877-025-06967-6)
Supplement: Supplementary file 3 — Additional file 3. Baseline characteristics of the participants per-protocol. [file 12877_2025_6967_MOESM3_ESM.docx]

**Additional file 4: Secondary results stratified for frailty status**

**Table 1. The mean scores of the Four Stage Balance Test, Timed Up and Go Test and the 36-Item Short Form Health Survey of the intervention group and control group stratified for frailty status.**

|  |  | **Intervention group  (mean (SE)) (n = 131)** | | |  | **Control group (mean (SE)) (n = 133)** | | |  | **RE crude analysis (95% CI)** | **RE crude analysis (95% CI)** | **RE crude analysis (95% CI)** | **RE adjusted for confounders^1^ (95% CI)** | **RE adjusted for confounders^1^ (95% CI)** | **RE adjusted for confounders^1^ (95% CI)** |
| --- | --- | --- | --- | --- | --- | --- | --- | --- | --- | --- | --- | --- | --- | --- | --- |
|  |  | **M0** | **M4** | **M12** |  | **M0** | **M4** | **M12** |  | **M0 – M4** | **M0 – M12** | **Overall effect** | **M0 – M4** | **M0 – M12** | **Overall effect** |
| Four stage Balance Test |  |  | | |  |  | | |  |  |  |  |  |  |  |
| *Non-frail* |  | 34.7 (1.05) | 35.7 (1.05) | 33.9 (1.26) |  | 35.7 (0.99) | 35.4 (1.55) | 35.4 (1.29) |  | 1.34 (-2.59; 5.28) | -0.46 (-4.19; 3.28) | 0.14 (-2.65; 2.93) | 1.34 (-2.59; 5.28) | -0.46 (-4.19; 3.28) | 0.14 (-2.65; 2.93) |
| *Pre-frail* |  | 34.6 (0.70) | 34.9 (0.85) | 31.7 (1.04) |  | 33.8 (0.70) | 33.0 (1.46) | 32.2 (1.34) |  | 1.07 (-2.41; 4.55) | -1.36 (-4.99; 2.27) | -0.55 (-3.18; 2.08) | 1.07 (-2.41; 4.55) | -1.36 (-4.99; 2.27) | -0.55 (-3.18; 2.08) |
| Timed Up and Go Test |  |  | | |  |  | | |  |  |  |  |  |  |  |
| *Non-frail* |  | 8.52 (0.35) | 8.00 (0.39) | 8.32 (0.36) |  | 8.25 (0.27) | 8.57 (0.72) | 9.02 (0.60) |  | -0.84 (-2.46; 0.78) | -0.98 (-2.41; 0.45) | -0.93 (-2.01; 0.15) | -0.84 (-2.46; 0.78) | -0.98 (-2.41; 0.45) | -0.93 (-2.01; 0.15) |
| *Pre-frail* |  | 9.39 (0.25) | 8.88 (0.37) | 9.24 (0.34) |  | 9.50 (0.28) | 10.3 (0.96) | 10.4 (0.69) |  | -1.28 (-3.36; 0.81) | -1.00 (-2.69; 0.69) | -1.09 (-2.38; 0.20) | -1.28 (-3.36; 0.81) | -1.00 (-2.69; 0.69) | -1.09 (-2.38; 0.20) |
| 36-Item Short Form  Health Survey  Physical functioning |  |  |  |  |  |  |  |  |  |  |  |  |  |  |  |
| *Non-frail* |  | 87.3 (1.78) | 87.5 (2.26) | 85.8 (2.41) |  | 82.5 (2.02) | 81.8 (3.91) | 82.0 (3.64) |  | 0.89 (-7.60; 9.38) | -1.00 (-9.37; 7.36) | -0.37 (-6.49; 5.74) | 0.89 (-7.60; 9.38) | -1.00 (-9.37; 7.36) | -0.37 (-6.49; 5.74) |
| *Pre-frail* |  | 67.1 (2.13) | 67.6 (4.14) | 67.6 (2.80) |  | 67.7 (2.06) | 64.7 (4.54) | 65.5 (4.62) |  | 3.58 (-9.86; 17.02) | 2.82 (-7.85; 13.49) | 3.07 (-5.06; 11.20) | 3.58 (-9.86; 17.02) | 2.82 (-7.85; 13.49) | 3.07 (-5.06; 11.20) |
| Emotional wellbeing |  |  | | |  |  | | |  |  |  |  |  |  |  |
| *Non-frail* |  | 86.8 (1.55) | 80.9 (2.10) | 84.6 (1.93) |  | 85.5 (1.66) | 82.7 (2.81) | 81.7 (3.36) |  | -3.15 (-9.82; 3.52) | 1.58 (-5.77; 8.92) | 0.00 (-5.27; 5.28) | -3.15 (-9.82; 3.52) | 1.58 (-5.77; 8.92) | 0.00 (-5.27; 5.28) |
| *Pre-frail* |  | 71.2 (1.53) | 73.6 (2.47) | 71.5 (2.49) |  | 69.8 (1.46) | 71.9 (3.16) | 65.8 (4.01) |  | 0.24 (-7.04; 7.51) | 4.29 (-5.70; 14.29) | 2.94 (-3.87; 9.76) | 0.24 (-7.04; 7.51) | 4.29 (-5.70; 14.29) | 2.94 (-3.87; 9.76) |

**RE = Relative Effect
^1^ Analysis adjusted for age and sex**
