# Supplementary material for: The effectiveness of a nation-wide implemented fall prevention intervention in the Netherlands in reducing falls and fall-related injuries among community-dwelling older adults with an increased risk of falls: a randomized controlled trial
Source: BMC Geriatr. 2026 Jan 24;26:227. doi: 10.1186/s12877-025-06967-6 (PMC12911379; doi:10.1186/s12877-025-06967-6)
Supplement: Supplementary file 4 — Additional file 4. Secondary results stratified for frailty status. [file 12877_2025_6967_MOESM4_ESM.docx]

**Additional file 1: Trial treatment manual of the In Balance fall prevention intervention**

**Intervention**

The In Balance intervention is a fourteen-week group programme for older adults at risk of falls and is provided by registered and certified physical therapists and exercise therapists (23). The aim of the intervention is to reduce falls by increasing awareness, balance and strength by combining educational and exercise components. The intervention consists of three phases. The first phase (week 1) comprises one information meeting about physical activity, the impact a fall can have on a person’s life and health, and the purpose of the In Balance intervention. In the second phase (week 2-4), there are three weekly educational meetings about increasing awareness of one’s fall risk and balance disturbance, increasing knowledge about how to implement effective fall prevention methods, and getting acquainted with the upcoming training weeks. The third phase (week 5-14) consists of a physical exercise programme with two one-hour training sessions per week. Exercises are derived from principles of Tai Chi, with balance and strength elements and with emphasis on standing strong and shifting weight. Education and Tai Chi exercises are known to be effective in reducing the incidence of falls (44-46). After each session, the participants receive homework, consisting of conducting exercises learned during the training sessions and reading parts of the textbook belonging to the In Balance intervention. Participants are expected to spend about one hour per week on this homework, distributed over several days. The 3 phases of the intervention are summarized in Figure 1.


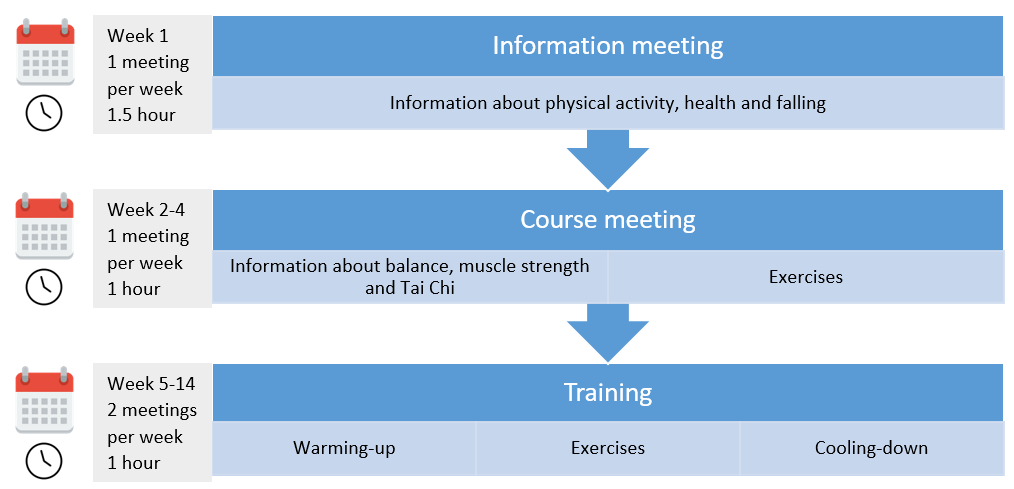


**Figure 1. Overview of the In Balance intervention**

**Control group**

The control group received written general physical activity recommendations in the form of a flyer. This flyer contained advice on physical activity levels, strength and balance for older adults. For example, a minimal amount of 150 minutes of moderate to vigorous physical activity per week was recommended, distributed over several days. Also muscle and bone strengthening activities such as walking stairs and balance exercises at least twice per week were advised. Moreover, the health benefits associated with physical activity were explained. These recommendations followed the Dutch Guidelines for Physical Activity (47).
