# Supplementary material for: The effectiveness of a nation-wide implemented fall prevention intervention in the Netherlands in reducing falls and fall-related injuries among community-dwelling older adults with an increased risk of falls: a randomized controlled trial
Source: BMC Geriatr. 2026 Jan 24;26:227. doi: 10.1186/s12877-025-06967-6 (PMC12911379; doi:10.1186/s12877-025-06967-6)
Supplement: Supplementary file 5 — Additional file 5. Secondary results per-protocol. [file 12877_2025_6967_MOESM5_ESM.docx]

**Additional file 5: Secondary results per-protocol**

**Table 1. The mean scores of the Four Stage Balance Test, Timed Up and Go Test and the 36-Item Short Form Health Survey of the intervention group and control group according to a per-protocol analysis.**

|  |  | **Intervention group  (mean (SE)) (n = 54)** | | |  | **Control group (mean (SE)) (n = 130)** | | |  | **RE crude analysis (95% CI)** | **RE crude analysis (95% CI)** | **RE crude analysis (95% CI)** | **RE adjusted for confounders^1^ (95% CI)** | **RE adjusted for confounders^1^ (95% CI)** | **RE adjusted for confounders^1^ (95% CI)** |
| --- | --- | --- | --- | --- | --- | --- | --- | --- | --- | --- | --- | --- | --- | --- | --- |
|  |  | **M0** | **M4** | **M12** |  | **M0** | **M4** | **M12** |  | **M0 – M4** | **M0 – M12** | **Overall effect** | **M0 – M4** | **M0 – M12** | **Overall effect** |
| Four stage Balance Test |  | 33.7 (0.97) | 35.2 (0.89) | 31.3 (1.19) |  | 34.3 (0.58) | 33.6 (1.24) | 33.0 (1.18) |  | 2.21 (-1.17; 5.60) | -1.12 (-4.42; 2.19) | -0.01 (-2.46; 2.44) | 2.21 (-1.17; 5.60) | -1.12 (-4.42; 2.19) | -0.01 (-2.46; 2.44) |
| Timed Up and Go Test |  | 9.54 (0.32) | 8.87 (0.38) | 9.09 (0.30) |  | 9.09 (0.22) | 9.80 (0.80) | 9.99 (0.56) |  | -1.38 (-3.23; 0.47) | -1.36 (-2.99; 0.27) | -1.37 (-2.60; -0.14) | -1.38 (-3.23; 0.47) | -1.36 (-2.99; 0.27) | -1.37 (-2.60; -0.14) |
| 36-Item Short Form  Health Survey  Physical functioning  Emotional wellbeing |  | 70.4 (2.70)  76.4 (2.05) | 72.8 (2.94)  77.3 (1.76) | 73.9 (2.62)  76.4 (1.95) |  | 71.9 (1.72)  74.1 (1.32) | 69.5 (4.03)  74.9 (2.71) | 70.2 (4.06)  70.1 (3.61) |  | 4.82 (-4.72; 14.36)   0.44 (-6.75; 7.64) | 5.21 (-4.55; 14.97)   4.42 (-4.30; 13.14) | 5.08 (-1.99; 12.15)   3.10 (-3.02; 9.21) | 4.82 (-4.72; 14.36)   0.44 (-6.75; 7.64) | 5.21 (-4.55; 14.97)   4.42 (-4.30; 13.14) | 5.08 (-1.99; 12.15)   3.10 (-3.02; 9.21 |

**RE = Relative Effect
^1^ Analysis adjusted for age and sex**
